# Real-World Experience with AGA Guidelines in the Management of Crohn's Disease following Ileocolonic Resection: A Retrospective Cohort Study

**DOI:** 10.1155/2020/8618574

**Published:** 2020-04-20

**Authors:** Shasha Tang, Wei Liu, Weilin Qi, Tunan Yu, Qian Cao, Xiaolong Ge, Wei Zhou

**Affiliations:** ^1^Department of General Surgery, Sir Run Run Shaw Hospital, School of Medicine, Zhejiang University, Hangzhou, China; ^2^Inflammatory Bowel Disease Center, Sir Run Run Shaw Hospital, School of Medicine, Zhejiang University, Hangzhou, China; ^3^Department of Gastroenterology, Sir Run Run Shaw Hospital, School of Medicine, Zhejiang University, Hangzhou, China

## Abstract

**Background:**

Postoperative endoscopic recurrence (PER) is common in patients with Crohn's disease (CD) after surgery. The impact of the American Gastroenterological Association (AGA) guideline adherence on PER in real life remains unclear.

**Methods:**

The postoperative management of CD patients undergoing ileocolonic resection with anastomosis from 2017 to 2018 was conducted based on the AGA guidelines. Colonoscopies were performed within one year after surgery. Clinical data and risk factors for endoscopic recurrence were analyzed focusing on postoperative pharmacological prophylaxis.

**Results:**

All patients were at a high risk of postoperative recurrence according to the AGA guidelines. PER occurred in 29 (28.7%) of these patients. The overall PER rate was 39.2% at one year. The PER rate in patients treated with nitroimidazole, thiopurines, infliximab, or a combination of thiopurines and infliximab for postoperative prophylaxis was 88.1%, 34.1%, 20.5%, and 0%, respectively. Cox regression showed that smoking at the time of surgery and AGA guideline adherence were independent factors associated with PER (HR: 3.75, 95% CI: 1.36-10.33, *P* = 0.01; HR: 0.36, 95% CI: 0.15-0.86, *P* = 0.02). In addition, further investigation revealed that educational background was the main factor related to patients' nonadherence to AGA guidelines.

**Conclusions:**

The majority of CD patients who undergo surgery in clinical practice may be at a high risk of disease recurrence. Thiopurines and infliximab are effective in preventing endoscopic recurrence. Guideline nonadherence is associated with PER at one year, thus indicating that there is room for improvement in adherence to the AGA guidelines.

## 1. Introduction

Crohn's disease (CD) is an idiopathic, chronic, relapsing, and formidable inflammatory disease that could affect the whole gastrointestinal tract, but the disease is predisposed to the ileocolon [1, 2]. Furthermore, the disease status of most patients may deteriorate from an inflammatory process into fibrostenotic and penetrating disease, which eventually leads to surgical intervention [3]. It has been reported that the risk of surgery in patients with CD at 1 and 5 years after diagnosis is 16% and 33%, respectively, and that approximately 70% of patients require surgical resection of the affected bowel segment during their lifetime [4–6].

Although surgery may ameliorate symptoms and improve the short-term quality of life in patients, it is not curative [4]. The majority of patients treated with ileocolonic resection experience postoperative recurrence. Several studies demonstrated that 30% to 85% of patients had PER at the neoterminal ileum proximal to the primary anastomosis within one year after the operation [6–8]. In addition, emerging research conducted by the American Gastroenterological Association (AGA) showed several risk factors, such as active smoking, age less than 30 years, and prior surgeries for penetrating disease, with or without perianal disease, that are correlated with postoperative recurrence [9]. A higher risk of recurrence is determined if patients have one of the above factors. Patients who are older than 50 years do not smoke and are undergoing their first surgery for a short segment of fibrostenotic disease (<10 to 20 cm) regarded as having a lower risk of recurrence. According to the AGA guidelines, prophylaxis with anti-TNF agents or thiopurines within 8 weeks of surgery is warranted for patients at a higher risk of disease recurrence, with a routine endoscopic follow-up examination to tailor therapy. In contrast, patients at a lower risk of recurrence should be scheduled a regular endoscopic follow-up examination starting 6-12 months after surgery if no prophylaxis was administered, allowing for early detection in the case of endoscopic recurrence.

In this study, we performed a retrospective analysis of postoperative endoscopic recurrence and management within one year after ileocolonic resection in CD patients. According to the AGA guidelines, postoperative prevention strategies were established to observe PER and identify risk factors for recurrence.

## 2. Materials and Methods

All patients with CD who underwent ileocolonic resection from January 2017 to June 2018 at the IBD Centre, Sir Run Run Shaw Hospital, School of Medicine, Zhejiang University, were identified. Patients with small bowel or colonic diversion, residual lesions, no endoscopic evaluation within one year after surgery, or a lack of detailed data were excluded. We considered the time of the final bowel continuity restoration as the first postoperative day if the surgical procedure involved multiple stages. All patients included signed informed consent forms, and the study was approved by the ethics committee of our hospital.

The baseline characteristics of the patients included sex, age at diagnosis, age at surgery, disease duration, active smoking at surgery, Montreal classification, concomitant perianal disease, history of surgery, preoperative medications (5-ASA, immunomodulator, steroid, and infliximab), postoperative complications, postoperative treatments, PER within one year, and adherence to the AGA guidelines.

Regarding prophylaxis for PER, thiopurines and infliximab are commonly regarded as potent drugs with or without metronidazole. In this study, thiopurines (1.0-1.5 mg/kg for 6-MP and 2.0-2.5 mg/kg for AZA) and infliximab (5 mg/kg) were administered based on the weight of the patents. PER was determined at the first colonoscopy performed within one year after surgery, and recurrence at the anastomosis site or new terminal ileum was defined as a Rutgeerts score ≥ i2 [8]. Colonoscopy was not performed at a specific time in patients in the study. Endoscopic recurrence was used as the primary endpoint.

According to the AGA guidelines, patients with risk factors, such as younger age (less than 30 years), smoking, and two prior surgeries for penetrating disease, with or without perianal disease, were regarded as having a higher risk of recurrence and were treated with thiopurines or anti-TNF agents for preventing recurrence. Accordingly, patients who were older than 50 years, smoked, and were undergoing their first surgery for a short segment of fibrostenotic disease (<10 to 20 cm) were regarded as having a lower risk of recurrence, and they were managed without therapy but with routine scheduled endoscopic follow-up examinations, starting 6-12 months after surgery, to detect endoscopic recurrence. Patients who did not comply with the AGA guidelines were regarded as nonadherent, and the primary reasons associated with nonadherence were determined by a follow-up telephone interview.

## 3. Statistical Analysis

Statistical Product and Service Solutions (SPSS) version 21.0 (Armonk, NY: IBM Corp) was used to analyze the data. Quantitative variables are described as the median and interquartile range. Qualitative variables are described as the frequency and percentage. The cumulative incidence of endoscopic recurrence and the incidence of endoscopic recurrence with different prophylactic treatments were calculated by the Kaplan-Meier method. The start date corresponded to the time of bowel continuity restoration. The identification of factors associated with recurrence was performed using bivariate Cox proportional hazards models. Parameters with a *P* value less than 0.1 on bivariate analysis were introduced into a Cox proportional hazards multivariable model. The results are expressed as the hazard ratio (HR) and 95% confidence interval (CI). Data were analyzed with SPSS 22.0 software. Statistical significance was considered at *P* < 0.05.

## 4. Results

### 4.1. Patient Characteristics

A total of 129 patients underwent ileocolonic resection with primary or secondary anastomosis for CD between January 2017 and June 2018, but only 101 of patients participated in endoscopic monitoring within one year after surgery. The remaining patients were lost to follow-up. Therefore, this cohort comprised 101 patients: 70 (68.3%) were males; the median age at diagnosis was 25 (20-31) years; the age at surgery was 29 (24-35) years; and the duration of disease was 48 (12-72) months. Thirteen (12.9%) patients were active smokers at the time of surgery; among them, 3 (23.1%) quit during the follow-up period. Thirty-seven (36.6%) and 18 (17.8%) patients had perianal disease and a history of appendectomy, respectively; 15 (14.8%) patients had a history of prior CD-related surgery. The indication for surgery was strictures in 47 (47.5%) patients, penetration in 35 (34.7%) patients, and inflammation in 18 (17.8%) patients. In terms of postoperative complications, 10 (9.9%) patients developed a surgical site infection, and 2 (2.0%) experienced abdominal abscess formation; all of them improved with conservative treatment. With respect to overall treatment before surgery for CD, 44.5% of patients had already received treatment with 5-ASA, 41.6% with immunomodulators, 23.8% with infliximab, and 31.8% with steroids. In addition, 18.8% of patients were preoperatively treated with a combination of infliximab and thiopurines. In all, 29.7% did not adhere to the AGA guidelines. The details of the demographic and clinical characteristics are shown in Table 1.

### 4.2. Postoperative Follow-Up

The AGA guidelines divide the risk categories for recurrence of CD after surgery into lower risk and higher risk. According to the above-described risk evaluation, all patients were at a higher risk of postoperative recurrence in the study. Among these patients, 29.7% only received treatment with metronidazole, 13.9% with infliximab, 41.6% with thiopurines, and 14.8% with a combination of thiopurines and infliximab simultaneously starting the first month after surgery.

**Figure 1:**
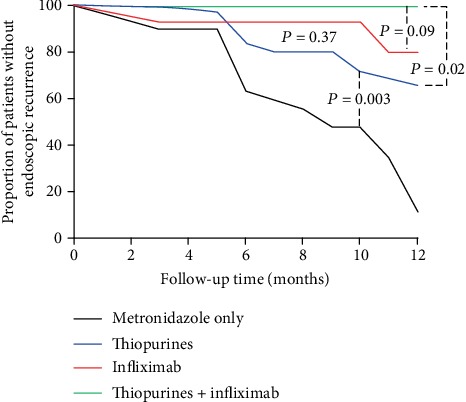
Kaplan-Meier curves for the proportions of endoscopic recurrence in patients treated with only metronidazole, thiopurines, infliximab, and combination of thiopurines and infliximab.

The median postoperative follow-up duration in the study was 7 (6–12) months. In all, 29 patients in our study developed endoscopic recurrence within one year after surgery. The overall PER rate was 39.2% at one year. The PER rate in patients treated with metronidazole alone, thiopurines, infliximab, and a combination of both thiopurines and infliximab for postoperative prophylaxis was 88.1%, 34.1%, 20.5%, and 0%, respectively. The results show that the PER rate in patients treated with thiopurines was lower than that in patients treated with metronidazole, with a significant difference (*P* = 0.003). The PER rate in patients treated with infliximab was also lower than that in patients treated with thiopurines, but there was no significant difference. Furthermore, the PER rate in patients treated with a combination of thiopurines and infliximab was significantly lower than that in patients treated with infliximab (*P* = 0.02; Figure 1).

### 4.3. Factors Associated with PER

The factors related to PER within one year are shown in Table 2. On univariate analysis, the stricture phenotype (HR = 0.36, 95% CI: 0.14-0.92, *P* = 0.03), active smoking (HR = 3.43, 95% CI: 1.48-7.91, *P* = 0.004), and adherence to the AGA guidelines (HR = 0.21, 95% CI: 0.09-0.47, *P* = 0.001) were significantly associated with PER. On multivariate analysis, active smoking (HR = 3.75, 95% CI: 1.36-10.33, *P* = 0.01) was a risk factor, and adherence to the AGA guidelines (HR = 0.36, 95% CI: 0.15-0.86, *P* = 0.02) showed an inverse association with PER.

### 4.4. Factors Related to Nonadherence to the AGA Guidelines

**Figure 2:**
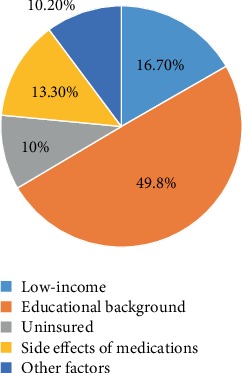
Factors related to nonadherence to the AGA guidelines.

By telephone follow-up interview, it was identified that educational background (49.8%) was the main factor associated with nonadherence to the AGA guidelines; patients without a high school diploma showed poor adherence compared with patients with a high school diploma (*P* = 0.03). Other factors, such as low income, uninsured status, and worry related to side effects, were also found to be associated with nonadherence to the guidelines (Figure 2).

## 5. Discussion

The present study was a real-world observational study of patients following ileocolonic resection, whose postoperative management was conducted based on the 2017 AGA guidelines [9]. Our study identified active smoking at the time of surgery and nonadherence to the AGA guidelines as independent predictors for PER. Adherence to the AGA guidelines was found to be associated with educational background. In addition, the study elucidated that thiopurines, infliximab, and combinations of both can be considered potent treatments in patients with a higher risk of recurrence.

In the current study, all patients were at a higher risk of recurrence. The overall PER rate within one year was 39.2%. Indeed, smoking was associated with PER, which is consistent with the findings of a previous study [10]. The other known risk factors for recurrence were not confirmed, apart from adherence to the guidelines. Although the gastroenterologist at our centre who claimed awareness and use of the guidelines in clinical practice had strongly advised the use of prophylaxis in patients with a higher risk of recurrence, 29.7% of patients did not adhere to the AGA guidelines. In general, the problem of nonadherence to treatment is often underestimated [11]. Prior studies indicated that nonadherence rates were higher among patients with chronic conditions than among those with acute conditions [12, 13]. It is well known that CD is a chronic and recurring disease that can affect the physical, mental, and psychological health of patients [14, 15]. Therefore, it is not surprising that approximately one-third of patients with CD are nonadherent to treatment [16].

Regarding the nonadherent patients, our study found that the barrier to complying with the AGA guidelines was mainly associated with educational background in most patients. Patients without a high school diploma showed poor adherence compared with patients with a high school diploma (*P* = 0.03). This might suggest the lack of understanding of the disease in patients with a low education level, even though a professional gastroenterologist provided adequate comprehensive guidance. A previous study conducted by Severs et al. also showed that altering the perception of an illness could be an approach to improve adherence behavior [17]. To some extent, other factors, such as an uninsured status, a low income, and anxiety related to the side effects of the medicine, also played a role in adherence to the guidelines. Several studies have also demonstrated that nonadherence to treatment may be associated with the disease duration, patient attitude towards treatment, medication cost, knowledge of the prescribed medication, and comprehension of the disease in the IBD population, consequently affecting the risk of relapse [18–21]. It is reasonable that physicians should pay more attention to patients' adherence to guidelines to prevent disease progression in patients with CD.

Our study shows that the PER rate in patients treated with nitroimidazole for postoperative prophylaxis was 88.1%. Nevertheless, prior studies have reported that the use of nitroimidazole for three months has great benefits in preventing short-term postoperative recurrence. A recent study also demonstrated that low-dose metronidazole reduces the endoscopic recurrence of CD postoperatively and that the intervention could be regarded as a therapeutic option in patients after surgery [22]. However, the high PER rate after treatment with only nitroimidazole in the patients in our study was not entirely unexpected because all patients were at a high risk of postoperative recurrence. Nitroimidazole alone is not recommended as prophylaxis for recurrence in these patients according to the AGA guidelines. In contrast, the studies mentioned above did not indicate the risk stratification of patients treated with nitroimidazole for postoperative recurrence. Thus, the overall benefit of the use of nitroimidazole in preventing postoperative CD recurrence still seems unclear. Further study may shed light on the justification of using nitroimidazole to prevent recurrence in lower-risk patients.

The PER rate after treatment with thiopurines, infliximab, and combinations of both was 34.1%, 20.5%, and 0%, respectively, indicating that combination treatment is superior to monotherapy for preventing postoperative recurrence. This result is in agreement with that of previous studies [23, 24]. One study reported that combination treatment is presumed to prevent immunogenicity against infliximab and increase its blood level, leading to a better long-term outcome [10]. No person who received combination treatment showed PER in our study within one year. The outcome was ideal, and it might be associated with the limited number of patients and the short follow-up time. Infliximab showed greater efficacy for the prevention of PER than thiopurines, although there was no significant difference. A prior study conducted by Regueiro et al. also showed that infliximab reduced the PER rate to 9% [25]. In the present study, 20.5% of patients treated with infliximab showed postoperative recurrence. The reason for the difference from Regueiro et al.'s study may be the complete ethnic homogeneity of each population and the limited sample size in the two studies. Moreover, our patients were at a high risk of recurrence, and the difference could also be due to high exposure to infliximab before surgery in some patients, resulting in the progressive loss of a response to infliximab because of the production of antidrug antibodies, in turn affecting the overall PER rate. It is debatable whether patients who lose their response to infliximab before surgery could continue to use infliximab as prophylaxis after surgery. Many studies have reported different views [26, 27].

It is critical to state the limitations of this study. First, it was a retrospective study. All patients who underwent ileocolonic resection were at a higher risk of postoperative recurrence according to the AGA guidelines. This may represent potential selection bias. Second, it must be acknowledged that the postoperative follow-up time was short. Because the primary endpoint of the study was endoscopic recurrence, it is possible for the efficacy of the relevant drugs in preventing recurrence to be a confounding factor in the study. Nonetheless, it is probably beneficial for treatment escalation to be guided by endoscopy results. Third, this was a single-centre and small-sample study, and our results show no PER in patients treated with combinations of infliximab and thiopurines; additionally, adherence to the AGA guidelines was a predictor of PER. The outcomes might have been influenced by a lack of statistical power. Thus, well-designed prospective studies should be performed to assess the results of the current study.

In conclusion, this study comprises real-world experience with AGA guidelines in the management of CD following ileocolonic resection, and its greatest contribution is highlighting the importance of adherence to the guidelines in the postoperative management of CD patients. The majority of CD patients who undergo ileocolic resection in clinical practice may be at a high risk of disease recurrence. Thiopurines and anti-TNF agents are effective in preventing the postoperative recurrence of CD. Despite well-known risk-based guidelines for postoperative prophylactic treatment, nearly one-third of high-risk patients choose only antibiotic treatment. Guideline nonadherence is associated with endoscopic recurrence within one year, thus indicating that there is room for improvement in adherence to the AGA guidelines.

## Figures and Tables

**Table 1:** Demographic and clinical characteristics of CD patients.

Characteristics	*n* (%)/IQR
Gender (male)	70 (68.3)
Age at diagnosis (years)	25 (20-31)
Age at surgery (years)	29 (24-35)
Disease duration (months)	48 (12-72)
Perianal disease (yes)	37 (36.6)
Active smokers (yes)	13 (12.9)
Disease behavior (Montreal classification)	
B1 nonstricturing, nonpenetrating	18 (17.8)
B2 stricturing	48 (47.5)
B3 penetrating	35 (34.7)
Medication before surgery	
5-ASA	45 (44.5)
Immunomodulator	42 (41.6)
Steroids	31 (30.7)
Infliximab	24 (23.8)
Appendix resection history	18 (17.8)
Prior surgery related to CD	15 (14.8)
Prophylactic medication after index surgery	
Only antibiotics	30 (29.7)
Thiopurines	42 (41.6)
Infliximab	14 (13.9)
Thiopurines+infliximab	15 (14.8)
Postoperative complication (yes)	12 (11.9)
Mean follow-up time (m)	7 (6-12)
Postoperative endoscopic recurrence	29 (28.7)

**Table 2:** Logistic regression analysis of postoperative endoscopic recurrence of CD.

	Univariate			Multivariate		
	HR	95% CI	*P*	HR	95% CI	*P*
Gender	0.54	0.22-0.34	0.21			
Age at diagnosis (Y)	0.83	0.23-2.96	0.89			
Disease duration (<48 M)	0.74	0.36-1.56	0.44			
Age at surgery (<29 Y)	1.54	0.74-3.21	0.25			
Disease phenotype (Montreal classification)						
B1	Reference					
B2	0.36	0.14-0.92	0.03	0.73	0.26-1.38	0.28
B3	0.51	0.20-1.31	0.16			
Perianal disease	0.85	0.39-1.82	0.67			
Prior surgery related to CD	0.65	0.19-2.16	0.48			
Active smokers	3.43	1.48-7.91	0.004	3.75	1.36-10.33	0.01
Preoperative medications						
5-ASA	Reference					
Azathioprine	0.70	0.33-1.52	0.37			
Steroid	1.26	0.59-2.70	0.55			
Infliximab	0.63	0.24-1.56	0.31			
Immunomodulator and IFX	1.61	0.71-3.63	0.25			
Appendix resection history	0.50	0.15-1.67	0.22			
Postoperative complication	1.3	0.31-5.65	0.70			
Adherence to AGA guideline	0.21	0.09-0.47	0.001	0.36	0.15-0.86	0.02

## Data Availability

The data used to support the findings of this study are available from the corresponding author upon request.
